# Evaluation of AI Chatbot Responses to a Standardized Patient Query on Myelin Oligodendrocyte Glycoprotein Antibody–Associated Disease: Cross-Sectional Content Analysis

**DOI:** 10.2196/81720

**Published:** 2026-04-29

**Authors:** Meryem Tuba Sönmez, Mehmet Fatih Yetkin, Duygu Arslan Mehdiyev, Nazlı Durmaz Çelik, Merve Bahar Ercan, Pınar Öztürk, Yeşim Eylev Akboğa, Emine Rabia Koç, Semra Mungan

**Affiliations:** 1 Neurology Clinic, Ankara Etlik City Hospital, Ankara, Türkiye; 2 Department of Neurology, Faculty of Medicine, Erciyes University, Gevher Nesibe Hospital, Prof. Dr. Turhan Feyzioğlu Street No. 42, Kayseri, Melikgazi, 38030, Türkiye, 90 352 2076666; 3 Department of Neurology, Eskişehir Osmangazi University, Eskişehir, Türkiye; 4 Department of Neurology, Gazi University, Ankara, Türkiye; 5 Neurology Clinic, Ankara Yıldırım Beyazıt University Yenimahalle Training and Research Hospital, Ankara, Türkiye; 6 Neurology Clinic, Sincan Research and Training Hospital, Ankara, Türkiye; 7 Department of Neurology, Bursa Uludag University, Bursa, Türkiye; 8 Department of Neurology, University of Health Sciences Ankara City Hospital, Ankara, Türkiye

**Keywords:** artificial intelligence, large language models, chatbots, patient education, myelin oligodendrocyte glycoprotein antibody–associated disease, readability, health information quality, citation transparency

## Abstract

**Background:**

Large language model–based chatbots are increasingly used by the public to access medical information. Although these tools can improve access and convenience, their quality, clarity, and transparency remain uncertain for rare and diagnostically complex neurological conditions, such as myelin oligodendrocyte glycoprotein antibody–associated disease (MOGAD).

**Objective:**

This study aimed to evaluate the scientific quality, understandability, citation transparency, and readability of responses generated by widely used artificial intelligence chatbot platforms to a standardized, patient-centered query on MOGAD.

**Methods:**

We conducted a cross-sectional content analysis using the query, “What is MOGAD, and how is MOGAD treated?” Ten widely accessible chatbot platforms were queried once on the same day in new sessions. Responses were anonymized and independently evaluated by 7 blinded neurologists using DISCERN (treatment-related scientific quality), Patient Education Materials Assessment Tool for Printable Materials (PEMAT-P), and the Web Resource Rating (WRR; citation transparency). Readability was assessed using the Flesch-Kincaid Grade Level (FKGL) and Coleman-Liau Index, and word count was recorded. Platforms were compared by functional orientation and the access model. Mann-Whitney *U* and Kruskal-Wallis tests with Dunn post hoc tests were used. Interrater reliability was assessed using intraclass correlation coefficients.

**Results:**

Significant differences were observed across platforms for DISCERN, PEMAT-P, and WRR scores (all *P*<.001). Search-focused platforms achieved higher understandability than conversation-focused platforms (median PEMAT-P 52.6, IQR 47.4-54 vs 46.7, IQR 42-47.3; *P*=.04), whereas conversation-focused platforms had higher WRR scores (median 26.8, IQR 19.6-26.8 vs 19.6, IQR 19.6-25.9; *P*=.001). DISCERN scores did not differ significantly by functional orientation (*P*=.11). Paid-access platforms outperformed free-access platforms in DISCERN (median 42, IQR 36-45 vs 33, IQR 23.8-41.3; *P*<.001), PEMAT-P (median 52.6, IQR 46-54 vs 46, IQR 26.3-47.4; *P*=.002), and WRR (median 26.8, IQR 23.2-26.8 vs 10.7, IQR 3.57-19.6; *P*<.001). However, no statistically significant differences were observed between paid and free platforms in response length (median word count 336, IQR 271-369 vs 206, IQR 116-294; *P*=.11) or readability metrics. FKGL scores were comparable between paid and free outputs (median 17.54, IQR 16.6-18.4 vs 17.56, IQR 16.5-17.6; *P*=.61), and Coleman-Liau Index values similarly showed no significant difference by access model (median 21.30, IQR 20.6-22.3 vs 21.71, IQR 20.9-22.1; *P*=.91). Readability remained limited: all outputs exceeded recommended public health readability thresholds (FKGL≥8). High interrater agreement was observed (intraclass correlation coefficient=0.902 for DISCERN, 0.887 for WRR, and 0.838 for PEMAT-P).

**Conclusions:**

Artificial intelligence chatbot responses to a patient-centered MOGAD query varied substantially in scientific quality, understandability, transparency, and readability. Search-focused systems were more understandable, whereas conversation-focused systems showed greater citation transparency. Paid-access platforms achieved higher quality and transparency scores, without differences in readability or response length. All outputs exceeded recommended public health readability thresholds. These findings highlight the need for context-sensitive evaluation of chatbot outputs in rare and clinically complex conditions such as MOGAD.

## Introduction

Artificial intelligence (AI) is rapidly reshaping health care, influencing how patients access, interpret, and interact with medical information. One of the most visible applications of this technology is the rise of large language models (LLMs), now widely available through chatbot interfaces. These systems can generate fluent, natural-sounding responses to health-related questions, offering the promise of more accessible and personalized communication. However, the consistency, accuracy, and clarity of their responses remain uncertain, especially when the topic involves rare or diagnostically challenging conditions [1-4].

Myelin oligodendrocyte glycoprotein antibody–associated disease (MOGAD) is one such condition. It is a rare, immune-mediated disorder of the central nervous system that can present in a variety of ways, including optic neuritis, transverse myelitis, and acute disseminated encephalomyelitis [5]. The publication of international diagnostic criteria in 2023 has improved the ability to distinguish MOGAD from related disorders such as multiple sclerosis and aquaporin-4 antibody-positive neuromyelitis optica spectrum disorder [6]. Despite this progress, clinical awareness of MOGAD remains limited, and many patients and caregivers turn to online resources in search of reliable information [7,8].

In this context, AI-powered chatbots have emerged as promising tools for bridging knowledge gaps, especially for people affected by rare diseases. These platforms can offer quick, conversational responses to health-related questions, simulating interaction with a knowledgeable guide. Yet most prior studies evaluating chatbot performance have focused on common medical conditions [9,10]. Very few have investigated how these tools respond to queries about complex and less-recognized diseases like MOGAD, and even fewer have used multidimensional, validated instruments to assess the quality, clarity, and transparency of the information provided [11,12]. Furthermore, studies involving blinded expert evaluations and structured comparisons across different chatbot platforms remain limited [13,14].

To address these gaps, this study systematically evaluated the responses of ten widely used AI chatbot platforms to a standardized, patient-centered query regarding MOGAD. Using established instruments, we assessed the quality, comprehensibility, citation transparency, and readability of each response. Chatbot performance was also compared based on access model (free or paid) and functional design (chat-based or search-based). All responses were independently evaluated by experienced neurologists who were blinded to the source of each answer. This study aims to provide insight into the reliability and educational value of chatbot-generated medical content in the context of rare diseases and to inform best practices for the safe and effective use of AI in digital health communication.

## Methods

### Study Design, Query Selection, and Chatbot Sampling

This cross-sectional content analysis was designed to evaluate the quality, readability, comprehensibility, and source transparency of medical information generated by AI chatbot platforms in response to a standardized, patient-centered query related to MOGAD. Only publicly accessible chatbot outputs were used, and no human participants, personal health information, or clinical interactions were involved.

To identify the most relevant public-facing search terms related to MOGAD, a 5-year global search trend analysis (from May 20, 2020, to May 20, 2025) was conducted using Google Trends. The term “MOGAD” exhibited a marked increase in popularity, reflecting growing public awareness. Among the top ten co-occurring search terms, those most directly related to the disease included: “mogad disease,” “mog,” “what is mogad,” “mogad treatment,” “mogad symptoms,” and “mogad radiology.” Other common terms such as “nmosd,” “ms,” “nmo,” and “adem” were excluded because they were not specific to MOGAD.

Based on these findings, a standardized, patient-oriented question was formulated to reflect a realistic information-seeking behavior of individuals affected by MOGAD. The final query selected for evaluation was: “What is MOGAD, and how is MOGAD treated?*”*

This question was submitted on May 24, 2025, to ten widely accessible AI chatbot platforms. These platforms were purposefully chosen to represent diversity in model architecture, developer affiliation, and access modality (ie, free vs paid). The chatbot platforms evaluated included Copilot (Microsoft), Claude 3.5 and Claude 3.7 (Anthropic), Perplexity Pro (Perplexity AI), Grok 3 (xAI), Gemini Advanced and Gemini 2.5 Pro (Google), ChatGPT-4 and ChatGPT-4.5 (OpenAI), and DeepSeek (DeepSeek AI).

For consistency and ecological validity, each chatbot was queried only once in a newly initiated session. No follow-up prompts, rephrased questions, or clarifications were used. During data collection, all internet-browsing and retrieval features embedded within chatbot interfaces (eg, “Browse with Bing” or comparable web access modes) were disabled. Responses were generated in newly initiated sessions using a single standardized prompt, without follow-up queries, external link clicks, or activation of plugins or tools, and the first unedited output was captured verbatim and included in the evaluation dataset.

### Data Collection and Blinding Procedures

The study team consisted of 9 individuals. Two experienced neurologists (MTS and MFY) were responsible for the study design. MTS conducted a standardized training session outlining the study rationale and the evaluation instruments, while MFY remained blinded throughout the assessment phase and served as one of the independent raters.

NDÇ, who was not involved in the study design or evaluation, submitted all chatbot queries and anonymized the resulting outputs by assigning random numeric codes (1-10). These coded responses were then distributed to the evaluators without revealing the originating platforms.

Seven neurologists (MTS, MFY, DAM, MBE, PÖ, YEA, and SM), all blinded to chatbot identities, independently evaluated the anonymized responses. This group included MFY and 6 additional experts, each with at least 5 years of experience in diagnosing and managing demyelinating disorders. MTS and NDÇ were not blinded because they were directly responsible for preparing, coding, and distributing the response dataset. For transparency and reproducibility, chatbot platform names are shown in Multimedia Appendix 1; however, these identifiers were removed from the materials provided to blinded expert raters during the scoring process.

### Evaluation Instruments

Each chatbot response was evaluated in its original, fully formatted, and verbatim form as generated at the time of data capture. DISCERN, Patient Education Materials Assessment Tool for Printable Materials (PEMAT-P), and WRR scoring were conducted exclusively on these unedited outputs. Validated instruments and standardized measures were applied across four domains:

1. Scientific quality: DISCERN Tool, a 16-item instrument with a 5-point Likert scale (range: 16‐80), used to evaluate clarity, risk-benefit balance, citation of evidence, and overall reliability of treatment-related information. Higher scores indicate higher quality [15].
2. Comprehensibility: PEMAT-P (understandability domain*)* developed by the U.S. Agency for Healthcare Research and Quality (AHRQ), this tool measures the clarity of language, sentence structure, layout, and logical flow of information. Scores are expressed as percentages. Although PEMAT-P items are scored binarily, the overall percentage score derived from the 7 expert ratings was used as a continuous variable for the calculation of interrater reliability (intraclass correlation coefficient [ICC]) [16].
3. Source transparency: Web Resource Rating (WRR) assesses citation practices, author disclosure, source traceability, and update recency. Final scores are expressed as percentages [17]. In this study, WRR scoring was applied to the response content as presented to the user at the time of data capture. References were evaluated based on their visibility to the reader, accessibility, and explicit association with specific statements in the response text. Both hyperlink-based references and text-based citations were eligible for scoring. Hyperlinks were recorded as accessible references, whereas text-based citations were evaluated according to whether the source could be clearly identified and traced by the reader.
4. Readability: Coleman-Liau Index (CLI) and Flesch-Kincaid Grade Level (FKGL). CLI is based on character and sentence length [18]; FKGL considers word syllables and sentence structure [19]. Both metrics estimate the US grade level required to understand the text. Scores <8 indicate content appropriate for general public health communication [12]. Word counts were also recorded. For readability analysis only, URL strings, embedded hyperlinks, and reference lists were computationally removed prior to calculating word count, CLI, and FKGL in order to prevent artificial inflation of character and word counts. This preprocessing step was applied solely for quantitative readability assessment and did not affect expert scoring or the evaluation dataset.

### Instrument Licensing and Accessibility

All instruments used, DISCERN, PEMAT-P, CLI, FKGL, and WRR, are freely accessible, open-source tools. DISCERN and PEMAT-P are provided by Oxford University and AHRQ, respectively. CLI and FKGL use publicly available formulas, and WRR is distributed under a Creative Commons license. This ensures transparency, reproducibility, and ethical adherence in the methodology.

### Chatbot Categorization by Function and Access

For subgroup-level comparisons, chatbot platforms were classified according to 2 independent parameters: functional orientation and access model.

Functional orientation was defined based on the primary design characteristics of the chatbots. Platforms emphasizing natural dialogue, contextual flow, and conversational interaction were categorized as conversation-focused and included ChatGPT-4, ChatGPT-4.5, Claude 3.5, Claude 3.7, and Grok 3. In contrast, platforms optimized for information retrieval, structured responses, and search-enhanced outputs were designated as search-focused, comprising Copilot, DeepSeek, Gemini Advanced, Gemini 2.5 Pro, and Perplexity Pro.

The access model was defined based on the user entry cost at the time of data collection. Chatbots available without subscription or payment were categorized as free-access platforms: ChatGPT-4, Claude 3.5, Copilot, and DeepSeek. Platforms requiring a subscription or premium tier to access advanced versions were classified as paid-access, including ChatGPT-4.5, Claude 3.7, Gemini Advanced, Gemini 2.5 Pro, Perplexity Pro, and Grok 3.

### Qualitative Assessment of Content Depth

In addition to quantitative scoring, the 7 expert raters provided structured qualitative notes regarding specific content gaps (eg, absence of specific drug classes and lack of clinical nuance) for each response. These observations were aggregated to identify recurring thematic deficiencies across platforms.

### Statistical Analysis

Descriptive statistics were used to summarize chatbot performance across all predefined evaluation domains. For each AI platform, median (IQR) values and full ranges were calculated for 3 primary quality metrics: DISCERN (evidence-based treatment information), PEMAT-P (understandability), and WRR (reference transparency). Additionally, readability and response length were assessed using CLI, FKGL, and total word count.

Chatbots were further stratified by functional orientation (conversation-focused vs search-focused) and access model (free vs paid). To compare performance across these subgroups, nonparametric inferential statistics were applied. Specifically, the Mann-Whitney *U* test was used to compare median scores for DISCERN, PEMAT-P, and WRR between the 2 subgroups in each classification. Given the small subgroup sample sizes (n=5 per group), exact 2-sided tests were performed to ensure accurate *P* value estimation. Differences among multiple chatbot platforms were assessed using the Kruskal-Wallis test. For statistically significant Kruskal-Wallis results, pairwise comparisons were performed using the Dunn post hoc test with Bonferroni correction to control for the family-wise error rate. A *P* value less than .05 was considered indicative of statistical significance for all comparisons.

To assess interrater reliability, ICCs were computed for the 3 primary metrics. DISCERN and WRR scores were treated as continuous variables. Although PEMAT-P items are scored binarily, the overall percentage score derived from the 7 expert ratings was used as a continuous variable for the calculation of ICC, using a 2-way random-effects model with absolute agreement (ICC [1,2]). ICC values were interpreted according to conventional thresholds: values less than 0.50 indicate poor reliability, 0.50‐0.75 indicate moderate reliability, 0.75‐0.90 indicate good reliability, and values equal to or greater than 0.90 indicate excellent reliability [20].

**Figure 1.**
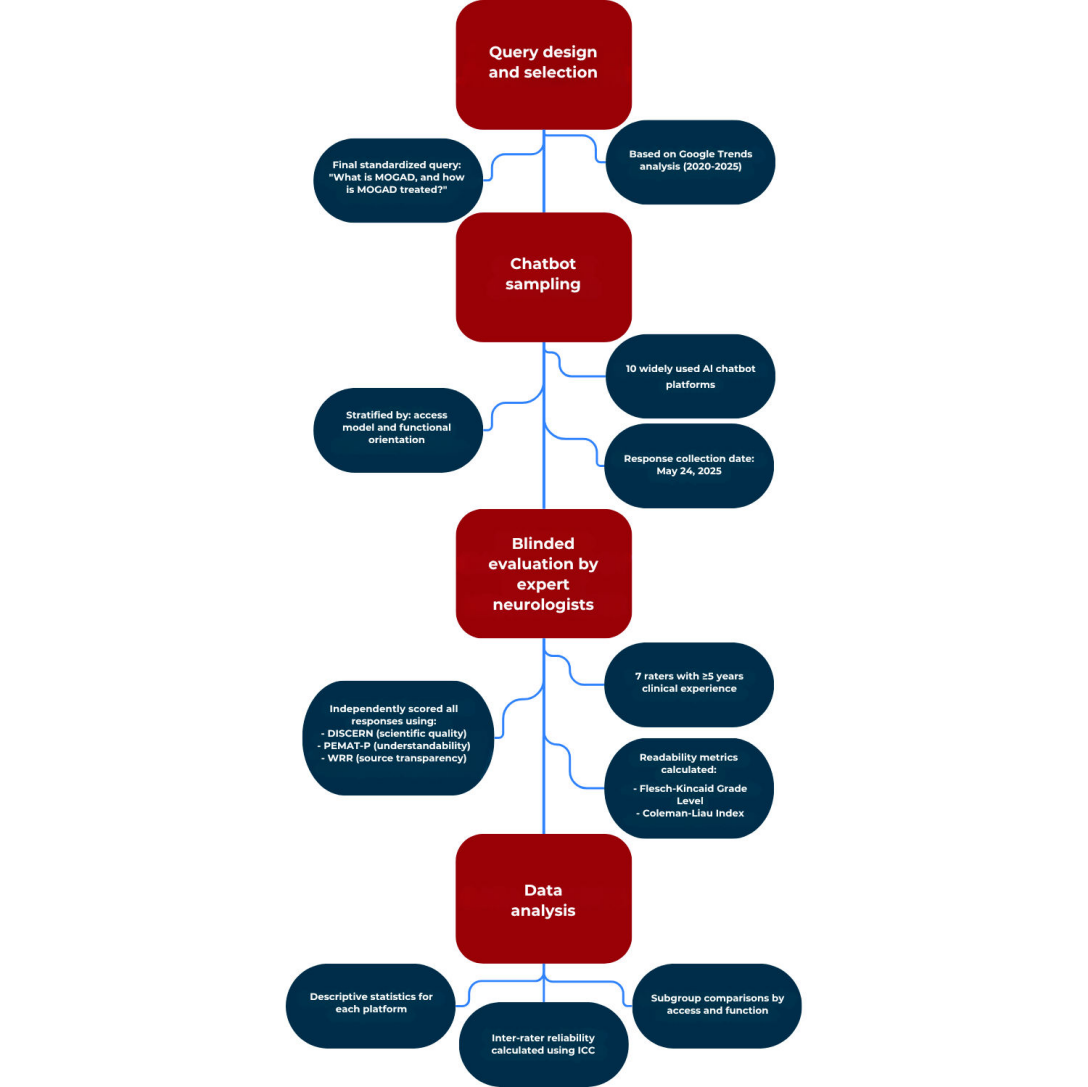
Stepwise workflow of the study evaluating artificial intelligence chatbot responses to a standardized myelin oligodendrocyte glycoprotein antibody–associated disease query. The process includes query design, chatbot selection, anonymized response collection, expert evaluation using validated tools, readability assessment, and statistical analysis. AI: artificial intelligence; ICC: intraclass correlation coefficient; MOGAD: myelin oligodendrocyte glycoprotein antibody–associated disease; PEMAT-P: Patient Education Materials Assessment Tool for Printable Materials; WRR: Web Resource Rating.

All statistical analyses were conducted using R software (version 4.4.3; R Core Team, R Foundation for Statistical Computing) and IBM SPSS Statistics (version 25.0). The study workflow is illustrated in Figure 1, which outlines the sequential methodology involving standardized query formulation, chatbot selection, anonymized response collection, blinded expert evaluation, and statistical analysis.

### Ethical Considerations

This study did not involve human participants, patient data, biological specimens, or any clinical intervention. All analyzed material consisted exclusively of publicly available, nonidentifiable text generated by AI chatbot platforms in response to a standardized informational query. The neurologists who served as blinded expert raters were not research participants, and no personal or identifiable information about them was collected, stored, or analyzed. Therefore, institutional ethics committee review and informed consent were not required. This determination is consistent with internationally recognized principles for research ethics, including the World Medical Association’s Declaration of Helsinki. In addition, under national research ethics regulations in Türkiye, ethical review is required primarily for studies involving identifiable human participants, human data, or clinical interventions; therefore, this study does not fall within the scope requiring institutional ethics approval [21].

## Results

### Overview of Chatbot Performance

Descriptive statistics for chatbot performance across key quality, readability, and transparency indicators are summarized in Table 1. Statistically significant differences were observed among platforms for DISCERN, PEMAT-P, and WRR scores (all *P*<.001, Kruskal-Wallis test).

**Table 1.** Descriptive statistics of chatbot responses regarding myelin oligodendrocyte glycoprotein antibody–associated disease information quality, readability, and transparency^a^.

Metric	ChatGPT-4	ChatGPT-4.5	Claude 3.7	Claude 3.5	Copilot	DeepSeek	Gemini 2.5 pro	Gemini advanced	Grok 3	Perplexity pro	*P* value
DISCERN^b^, median (IQR)	38 (38-41)	36 (33-38)	36 (34-36)	24 (23.5-29)	23 (20-24.5)	48 (43.5-52)	45 (43-45.5)	44 (43-44.5)	50 (48-52)	40 (39-44)	<.001
PEMAT-P^c^, median (IQR)	47.3 (46.7-47.3)	46 (46-47.3)	46 (42-50.7)	33 (29.7-33)	17 (17-21.7)	52.6 (50-54)	54 (53.3-58.5)	63 (57.8-63)	46 (46-50)	52.6 (52.6-53.3)	<.001
WRR^d^, median (IQR)	23.2 (19.6-26.8)	23.2 (21.5-26.8)	26.8 (26.8-26.8)	10.7 (3.6-10.7)	2.6 (3.6-3.6)	19.6 (19.6-19.6)	23.2 (19.6-25)	23.2 (21.4-23.2)	32.1 (26.8-32.1)	26.8 (26.8-28.5)	<.001
Word count	293	260	210	110	118	295	369	419	369	303	—^e^
CLI^f^	21.42	21.10	23.16	22.41	19.22	22.00	22.53	21.49	20.23	20.48	—
FKGL^g^	17.62	16.90	18.59	17.50	13.47	17.63	18.17	18.54	16.54	16.55	—

a Values are presented as median (IQR) for DISCERN, Patient Education Materials Assessment Tool for Printable Materials, and Web Resource Rating, and as single values for readability indices and word count. Chatbot evaluation comparisons for applicable variables across independent groups were performed using the Kruskal-Wallis test.

b DISCERN indicates the quality of treatment-related information.

c PEMAT-P: Patient Education Materials Assessment Tool for Printable Materials.

d WRR: Web Resource Rating.

e Not applicable.

f CLI: Coleman-Liau Index.

g FKGL: Flesch-Kincaid Grade Level.

DISCERN scores, reflecting the quality of treatment-related information, demonstrated notable variability across chatbots. Grok 3 exhibited the highest median DISCERN score (median 50, IQR 48-52), followed by DeepSeek (median 48, IQR 43.5-52) and Gemini 2.5 Pro (median 45, IQR 43-45.5). Intermediate median scores were observed for ChatGPT-4 (median 38, IQR 38.0-41), ChatGPT-4.5 (median 36, IQR 33-38), and Claude 3.7 (median 36, IQR 34-36). In contrast, lower median DISCERN scores were recorded for Claude 3.5 (median 24, IQR 23.5-29) and Copilot (median 23, IQR 20-24.5).

Understandability, as assessed using the PEMAT-P, varied across chatbots, with median scores ranging from 17 (IQR 17-21.7) for Copilot to 63 (IQR 57.8-63) for Gemini Advanced. Among free-access platforms, Copilot exhibited the lowest median PEMAT-P score (median 17, IQR 17-21.7), followed by Claude 3.5 (median 33, IQR 29.7-33).

WRR scores, reflecting citation transparency, varied across chatbots. The highest median WRR values were observed for Grok 3 (median 32.1, IQR 26.8-32.1), Claude 3.7 (median 26.8, IQR 26.8-26.8), and Perplexity Pro (median 26.8, IQR 26.8-28.5). In contrast, lower median WRR scores were recorded for Copilot (median 3.6, IQR 3.6-3.6) and Claude 3.5 (median 10.7, IQR 3.6-10.7).

In terms of readability, CLI values ranged from 19.22 (Copilot) to 23.16 (Claude 3.7), while FKGL scores varied from 13.47 (Copilot) to 18.59 (Claude 3.7). All FKGL values exceeded the recommended Grade 8 threshold for public health communication. Word count ranged from 110 words (Claude 3.5) to 419 words (Gemini Advanced).

**Figure 2.**
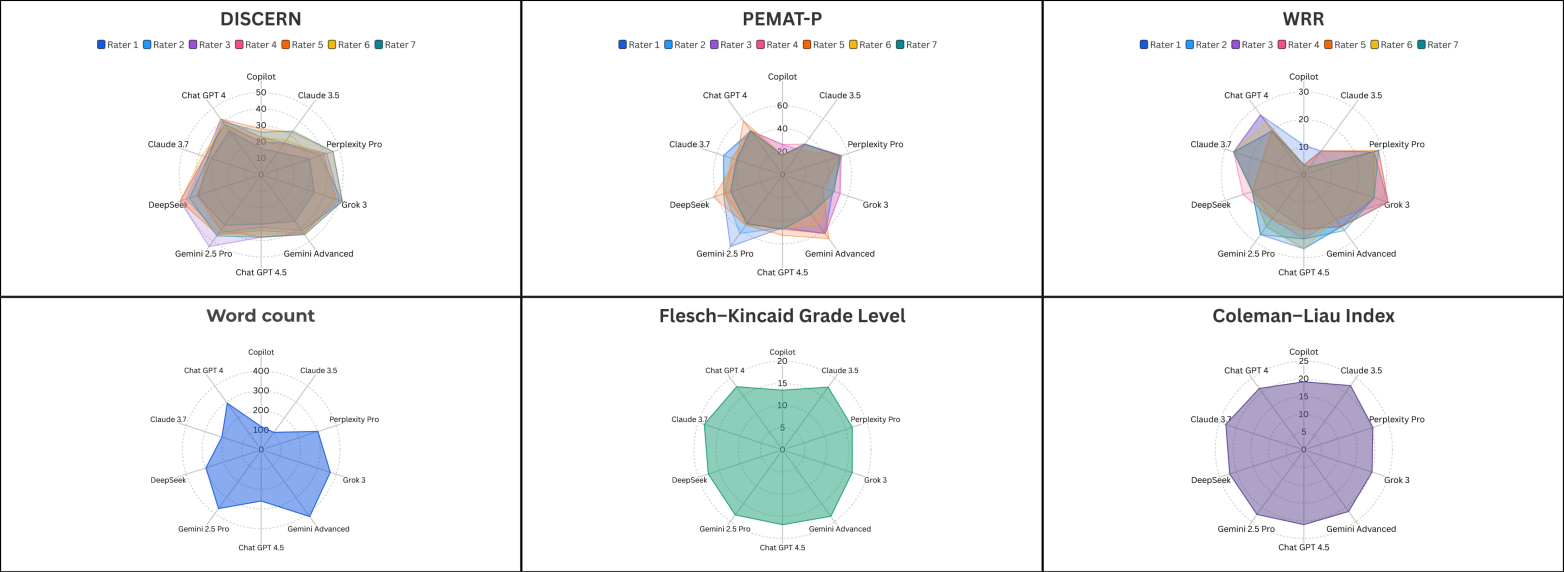
Multidimensional performance profiles of ten artificial intelligence chatbot platforms in response to a standardized myelin oligodendrocyte glycoprotein antibody–associated disease query. Radar plots illustrate chatbot performance across six key metrics: DISCERN (scientific quality), Patient Education Materials Assessment Tool for Printable Materials (understandability), Web Resource Rating (source transparency), word count (response length), Flesch-Kincaid Grade Level, and Coleman-Liau Index (readability). The top row shows interrater variability for expert-rated metrics (DISCERN, Patient Education Materials Assessment Tool for Printable Materials, and Web Resource Rating), while the bottom row presents readability and length scores derived from single-response outputs. PEMAT-P: Patient Education Materials Assessment Tool for Printable Materials; WRR: Web Resource Rating.

A visual summary of each chatbot’s multidimensional performance across 6 metrics is provided in Figure 2, illustrating differences in content quality, transparency, readability, and response length.

**Figure 3.**
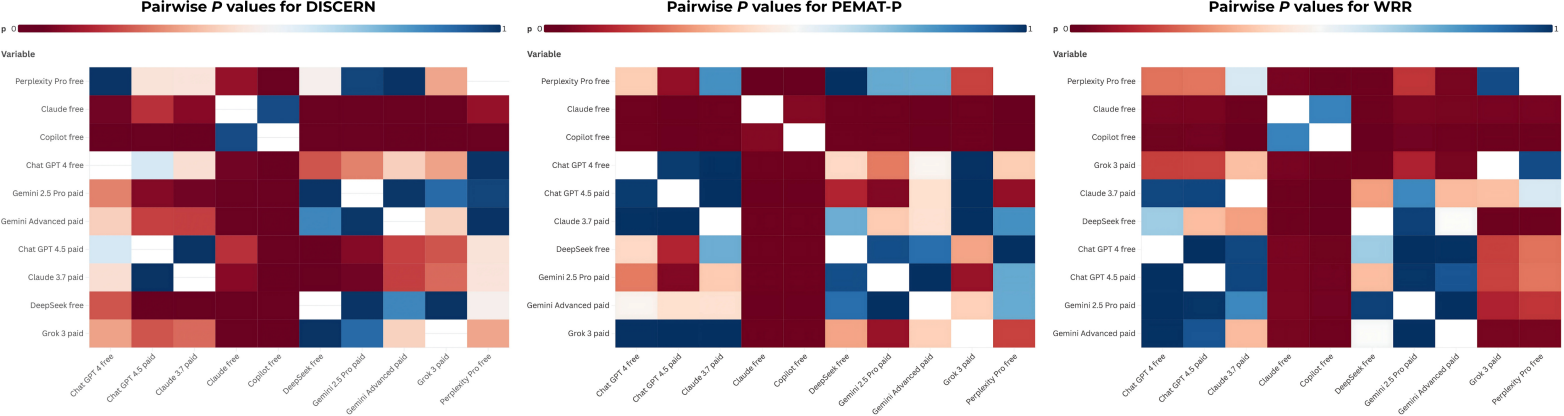
Pairwise statistical comparisons of ten artificial intelligence chatbot platforms across three expert-rated performance metrics (DISCERN, Patient Education Materials Assessment Tool for Printable Materials, and Web Resource Rating). This figure presents three separate heatmaps illustrating the results of the Dunn post hoc test with Bonferroni correction, which was applied following the overall significant Kruskal-Wallis test results (*P*<.001) across the ten evaluated chatbot platforms (n=10 platforms, assessed by seven independent expert neurologists). The heatmaps compare all possible pairs of the ten platforms based on their median scores for DISCERN (scientific quality of treatment information), Patient Education Materials Assessment Tool for Printable Materials (understandability), and Web Resource Rating (source transparency). The X-axis and Y-axis represent the ten unique artificial intelligence chatbot platforms compared in this study. The color intensity in each cell represents the adjusted *P* value of the pairwise comparison. Deep red tones (higher *P* values) indicate no statistically significant difference between the two compared platforms (*P*>.05), while deep blue tones (lower *P* values) denote a statistically significant difference in performance (*P*<.05). These heatmaps are essential for identifying the specific platform pairings responsible for the high overall performance variability observed in the expert-rated metrics. PEMAT-P: Patient Education Materials Assessment Tool for Printable Materials; WRR: Web Resource Rating.

Figure 3 displays pairwise comparisons using the Dunn post hoc test following Kruskal-Wallis analysis, further emphasizing the performance variability among platforms.

**Figure 4.**
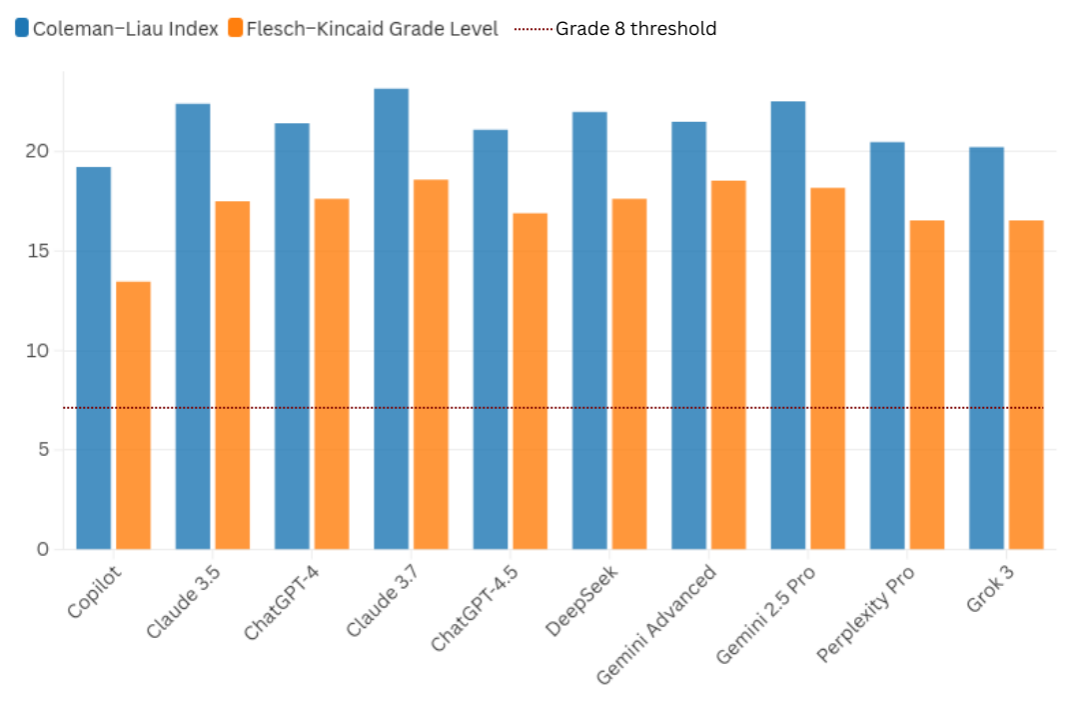
Combined readability scores of chatbot responses based on Coleman-Liau Index and Flesch-Kincaid Grade Level. Comparative readability scores for the ten artificial intelligence platforms using Coleman-Liau Index (blue bars) and Flesch-Kincaid Grade Level (orange bars). Scores are plotted against the recommended maximum Grade 8 threshold (red dashed line) for public health communication. CLI: Coleman-Liau Index; FKGL: Flesch-Kincaid Grade Level.

The comparative readability assessment of responses generated across the 10 evaluated AI chatbot platforms is graphically presented in Figure 4.

### Comparison Between Conversation-Focused and Search-Focused Chatbots

Performance metrics stratified by chatbot functionality are summarized in Table 2. Although search-focused models showed higher median DISCERN scores (median 44, IQR 36.5-45.5) than conversation-focused models (median 38, IQR 32.5-39), this difference did not reach statistical significance (*P*=.11).

**Table 2.** Comparison of quality, readability, and transparency metrics by functionality^a^.

Metric	Conversation-focused, median (IQR)	Search-focused, median (IQR)	*P* value
DISCERN	38 (32.5-39)	44 (36.5-45.5)	.11
PEMAT-P^b^	46.7 (42-47.3)	52.6 (47.4-54)	.04
WRR^c^	26.8 (19.6-26.8)	19.6 (19.6-25.9)	.001
Word count	260 (210-293)	303 (295-369)	.21
CLI^d^	21.42 (21.10-22.41)	21.49 (20.48-22)	.75
FKGL^e^	17.50 (16.90-17.62)	17.63 (16.55-18.17)	.92

a Values are presented as median (IQR). DISCERN refers to the quality of treatment-related information. Patient Education Materials Assessment Tool for Printable Materials indicates understandability. Web Resource Rating reflects transparency and citation of external sources. The Coleman-Liau Index measures textual complexity; higher scores indicate more complex language. Flesch-Kincaid Grade Level represents the U.S. school grade level required to comprehend the text; higher scores denote reduced accessibility for the general public. Conversation-focused chatbots: ChatGPT-4, ChatGPT-4.5, Claude 3.7, Claude 3.5, Grok 3. Search-focused chatbots: Gemini Advanced, Gemini 2.5 Pro, Perplexity Pro, Copilot, DeepSeek. Group comparisons were performed using the Mann-Whitney *U* test.

b PEMAT-P: Patient Education Materials Assessment Tool for Printable Materials.

c WRR: Web Resource Rating.

d CLI: Coleman-Liau Index.

e FKGL: Flesch-Kincaid Grade Level.

Search-focused platforms also showed higher median PEMAT-P scores compared with conversation-focused platforms (median 52.6, IQR 47.4-54 vs 46.7, IQR 42.0-47.3). Although this difference reached statistical significance (*P*=.04), the small subgroup sample size (n=5 per group) warrants cautious interpretation. In contrast, WRR scores were higher among conversation-focused platforms than search-focused platforms (median 26.8, IQR 19.6-26.8 vs 19.6, IQR 19.6-25.9; *P*=.001).

Median word count was greater among search-focused platforms (median 303, IQR 295-369) than conversation-focused platforms (median 260, IQR 210-293), but this difference was not statistically significant (*P*=.21).

No statistically significant differences were observed between functional orientations for readability metrics, including CLI (median 21.49, IQR 20.48-22 vs 21.42, IQR 21.10-22.41; *P*=.75) and Flesch-Kincaid Grade Level (median 17.63, IQR 16.55-18.17 vs 17.50, IQR 16.90-17.62; *P*=.92).

### Comparison Between Paid and Free Chatbots

Chatbots were also compared based on their access model, distinguishing between paid-access platforms and free-access platforms. Comparative performance metrics are summarized in Table 3.

**Table 3.** Comparison of quality, readability, and transparency metrics by access type^a^.

Metric	Paid, median (IQR)	Free, median (IQR)	*P* value
DISCERN	42 (36-45)	33 (23.8-41.3)	<.001
PEMAT-P^b^	52.6 (46-54)	46 (26.3-47.4)	.002
WRR^c^	26.8 (23.2-26.8)	10.7 (3.57-19.6)	<.001
Word count	336 (271-369)	206 (116-294)	.11
CLI^d^	21.30 (20.6-22.3)	21.71 (20.9-22.1)	.91
FKGL^e^	17.54 (16.6-18.4)	17.56 (16.5-17.6)	.61

a Values are presented as median (IQR). DISCERN reflects the quality of evidence-based treatment information. Patient Education Materials Assessment Tool for Printable Materials indicates understandability based on the Patient Education Materials Assessment Tool. Web Resource Rating assesses transparency and use of external references. The Coleman-Liau Index evaluates textual complexity; higher values indicate more difficult language. Flesch-Kincaid Grade Level denotes the U.S. school grade level required for comprehension; higher scores correspond to decreased readability. Paid chatbots: Perplexity Pro, ChatGPT-4.5, Claude 3.7, Gemini Advanced, Gemini 2.5 Pro, Grok 3. Free chatbots: ChatGPT-4, Claude 3.5, Copilot, DeepSeek. Group comparisons were performed using the Mann-Whitney *U* test.

b PEMAT-P: Patient Education Materials Assessment Tool for Printable Materials.

c WRR: Web Resource Rating.

d CLI: Coleman-Liau Index.

e FKGL: Flesch-Kincaid Grade Level.

Overall content quality, as assessed by the DISCERN total score was significantly higher among paid chatbots than free chatbots (median 42, IQR 36-45 vs 33, IQR 23.8-41.3, respectively; *P*<.001). Similarly, understandability scores measured using the PEMAT-P tool were greater in the paid group (median 52.6, IQR 46-54) compared with the free group (median 46, IQR 26.3-47.4; *P*=.002).

Transparency, evaluated using the WRR, differed markedly between access models. Paid chatbots achieved substantially higher WRR scores than free chatbots (median 26.8, IQR 23.2-26.8 vs 10.7, IQR 3.57-19.6; *P*<.001), reflecting more frequent inclusion of external references and source information.

Median word count did not differ significantly between access models, although paid chatbots generated numerically longer responses than free chatbots (median 336, IQR 271-369 vs 206, IQR 116-294; *P*=.11).

For readability metrics, no statistically significant differences were observed between paid and free platforms. FKGL scores were comparable between paid and free platforms (median 17.54, IQR 16.6-18.4 vs 17.56, IQR 16.5-17.6; *P*=.61). Similarly, CLI values did not differ significantly between groups (median 21.30, IQR 20.6-22.3 vs 21.71, IQR 20.9-22.1; *P*=.91).

The comparative strengths and limitations of each AI chatbot platform evaluated in this study are summarized in Table 4, highlighting the variability in treatment quality, citation transparency, comprehensibility, and readability across different models.

**Table 4.** Summary of strengths and limitations of artificial intelligence chatbot platforms in response to a standardized myelin oligodendrocyte glycoprotein antibody–associated disease-related patient query based on DISCERN, Patient Education Materials Assessment Tool for Printable Materials, Web Resource Rating, Flesch-Kincaid Grade Level, and Coleman-Liau Index assessments.

AI^a^ chatbots	Strengths	Limitations
ChatGPT-4	Freely accessible; offers moderate citation visibility (WRR^b^); designed for conversational interaction	Demonstrates only moderate performance in treatment quality (DISCERN); suboptimal understandability; exceeds recommended readability thresholds (FKGL^c^ >8)
ChatGPT-4.5	Strong citation practices; consistently high-quality content regarding treatment information (DISCERN)	Textual density compromises accessibility; readability metrics exceed public health communication standards
Claude 3.5	Open-access platform; conversationally fluent interface	Among the lowest in treatment quality (DISCERN) and source transparency (WRR); limited medical detail and citation support
Claude 3.7	Integrates citations effectively; maintains coherent conversational flow	Despite improved content structure, textual complexity remains high; readability concerns persist
Grok 3	Highest overall performance across DISCERN and WRR; provides comprehensive and well-referenced responses	Subscription required; lengthy and lexically dense outputs may reduce usability for non-expert audiences
Perplexity Pro	Search-augmented design; uses structured formatting (eg, bullet points); relatively strong performance in understandability	Exhibits high linguistic complexity (FKGL and CLI)^d^; demonstrates RAG^e^ errors, including acronym confusion, resulting in the inclusion of clinically irrelevant citations
Gemini Advanced	Achieved the highest score in understandability (PEMAT-P)^f^; benefits from search-enhanced architecture	Moderate citation transparency; content complexity remains above recommended readability thresholds
Gemini 2.5 Pro	Search-optimized structure; favorable DISCERN performance; clearly organized responses	Dense language and elevated FKGL scores limit accessibility; moderate citation performance
Copilot	Freely accessible; concise responses	Performed lowest in both understandability (PEMAT-P) and transparency (WRR) in this study; superficial content
DeepSeek	Freely accessible; balanced performance profile; favorable DISCERN scores; search-focused output generation	Moderate understandability and citation transparency; readability metrics exceed recommended limits for patient education

a AI: artificial intelligence.

b WRR: Web Resource Rating.

c FKGL: Flesch-Kincaid Grade Level.

d CLI: Coleman-Liau Index.

e RAG: retrieval-augmented generation.

f PEMAT-P: Patient Education Materials Assessment Tool for Printable Materials.

### Interrater Reliability Analysis

To assess the consistency of expert evaluations across chatbot-generated outputs, interrater reliability was evaluated using ICC. A 2-way random-effects model with absolute agreement (ICC [1,2]) was applied for continuous scores. The ICC for DISCERN total scores was 0.902, indicating excellent agreement among evaluators. Similarly, WRR scores demonstrated strong reliability with an ICC of 0.887. The PEMAT-P understandability scores also showed a high level of consistency, yielding an ICC of 0.838. These results suggest robust interrater agreement across all evaluated dimensions.

These findings provide a detailed snapshot of current chatbot performance across multiple dimensions and serve as a foundation for interpreting their implications in the broader context of digital health communication.

## Discussion

### Principal Findings

To the best of our knowledge, this is the first study to systematically evaluate chatbot-generated responses on MOGAD using a validated, multidimensional assessment framework. Across ten widely used platforms, we found statistically significant differences in scientific quality, understandability, and source transparency as measured by DISCERN, PEMAT-P, and WRR (*P*<.001 for all instruments). These differences were evident both between paid and free models and among chatbots with similar functional orientations. Paid-access platforms generally demonstrated higher scientific quality and citation transparency. Grok 3, a subscription-based conversational system, consistently produced the highest scores, whereas free-access platforms such as Copilot and Claude 3.5 showed lower performance. However, performance was not determined solely by access model, and marked individual variability across platforms was evident. Importantly, all chatbots generated content that exceeded recommended public health readability thresholds according to FKGL and CLI metrics, indicating a uniformly high level of linguistic complexity across platforms. No statistically significant differences were observed between paid and free platforms in readability (FKGL and CLI) or response length. Interrater reliability among the 7 blinded neurologists was high (ICC=0.902 for DISCERN, 0.887 for WRR, and 0.838 for PEMAT-P), supporting the robustness of our evaluation protocol.

Taken together, these results demonstrate marked variability in the clarity, scientific reliability, and source transparency of AI-generated medical information on MOGAD. Notably, improvements in scientific quality and transparency did not consistently translate into greater readability or easier comprehension for general audiences. Given that patients and caregivers may rely on such tools when navigating a rare and clinically complex neuroimmunological disorder, the observed differences across platforms underscore the importance of systematically evaluating chatbot outputs before integrating them into patient-facing contexts.

### Comparison With Prior Work

The performance differences we observed between paid and free access models are largely consistent with prior research. In our study, subscription-based platforms produced higher scores in scientific quality (DISCERN, *P*<.001), understandability (PEMAT-P, *P*=.002), and source transparency (WRR, *P*<.001). These findings align with studies reporting that premium systems tend to deliver more comprehensive and clinically aligned health information [12,22,23]. Such advantages may reflect differences in model architecture, update frequency, and overall technological capacity in favor of paid platforms [9].

However, higher cost does not necessarily translate into superior clarity or usability. Previous research has shown that some free access models can perform strongly, particularly in common and well-determined clinical topics. A striking example is found in the work of Şahin et al [13], who demonstrated that Copilot, the lowest scoring model across all metrics in our study, achieved the highest DISCERN score in an independent evaluation of erectile dysfunction content. This contrast illustrates how strongly chatbot performance can depend on clinical context and the formulation of the question.

Topic-dependent variability is a recurring theme in broader literature. Carlson et al [10] reported a wide range of performance across chatbots responding to vasectomy-related queries, with no platform demonstrating consistent superiority. Similar heterogeneity in accuracy, clarity, and transparency has been documented across studies involving sarcoidosis, chronic rhinosinusitis, appendicitis, benign anorectal disorders, and dental implantology [11,23-26]. In line with these observations, our analysis shows that variability becomes even more pronounced in MOGAD, a rare and phenotypically heterogeneous neuroimmunological disorder. The limited availability of high-quality open-access resources on MOGAD may further contribute to narrower or less comprehensive responses generated by certain models.

Across published evaluations of AI chatbots, a model’s functional orientation plays a central role in shaping the structure, clarity, and reliability of the medical information it produces. Studies conducted in areas such as sarcoidosis, chronic rhinosinusitis, appendicitis, and dental implantology consistently show that search-focused systems tend to generate more structured and easily navigable content that incorporates bullet points, subheadings, and hierarchical formatting [11,23-25]. In contrast, conversation-focused models typically provide a smoother narrative flow and stronger contextual coherence, allowing users to follow complex medical explanations more intuitively [13,24,27]. Our findings reflect this broader pattern: search-oriented platforms achieved higher PEMAT-P scores, while conversational systems demonstrated stronger WRR performance by integrating citations in a manner that was more consistent with the surrounding text.

Previous research also highlights a recurring vulnerability among search-focused platforms that rely on retrieval-augmented generation. Although these models often supply a larger number of citations, their references may not consistently align with the clinical context, and some may be only partially related to the queried topic [28]. In our study, this limitation was most clearly illustrated by Perplexity Pro, which achieved high WRR scores yet produced retrieval-related errors, including acronym confusion between “MOGAD” and unrelated terms such as “Monad.” This resulted in the inclusion of clinically irrelevant sources, including pharmaceutical products and conditions unrelated to neuroimmunology.

This finding underscores an important methodological limitation of the WRR instrument when applied to AI-generated content. WRR captures the visibility and quantity of cited web resources, but it does not assess the clinical appropriateness or accuracy of those citations. As a result, high transparency scores may coexist with retrieval errors that carry potential patient safety implications, particularly in search-based chatbots.

Conversely, while conversation-focused systems tend to produce more coherent narratives, they may be more prone to hallucinating mechanistic or treatment-related details when faced with topics that are uncertain or poorly represented in the literature [29]. Collectively, these findings indicate that each functional orientation carries distinct strengths and limitations. For rare and clinically heterogeneous conditions such as MOGAD, effective patient communication may require a balanced approach that draws on the structural advantages of search-focused models and the contextual strengths of conversational systems.

In our study, readability emerged as a consistent limitation across all platforms. As illustrated in Figure 4, every chatbot generated FKGL and CLI scores exceeding the recommended Grade 8 threshold for public health communication, indicating that the linguistic complexity of these outputs may pose challenges for users with limited health literacy. Similar findings have been reported in previous evaluations, where chatbot-generated medical information frequently exceeded recommended reading levels [11,12,23,24,27]. Taken together, the evidence suggests that elevated readability demands constitute a structural characteristic of current language models rather than a condition-specific exception.

For MOGAD, the implications of this burden are likely amplified. As a rare and clinically heterogeneous neuroimmunological disorder, MOGAD requires nuanced explanations of diagnostic criteria, relapse patterns, and treatment approaches, which may already be difficult even for medically literate users. When this level of clinical complexity is expressed through dense vocabulary and abstract phrasing, the cognitive load on patients and caregivers increases substantially. This interaction between disease complexity and high readability demands may heighten the risk of misunderstanding, false reassurance, or overly simplified self-directed interpretations. Ensuring that chatbot-generated information is linguistically accessible, therefore, remains essential for improving the safety and usability of AI-based medical communication in neuroimmunology.

### Limitations

This study has several limitations. First, the analysis was based on a single standardized patient query, which strengthened internal validity but limited generalizability to the more varied and interactive ways patients seek information in real-world settings. Second, only English-language outputs were evaluated, so performance in other languages or multilingual models remains uncertain. Third, chatbot responses were collected on a single day to minimize temporal drift, yet LLMs evolve rapidly, and their performance may change with future updates. Fourth, although validated instruments such as DISCERN, PEMAT-P, and WRR provided a structured basis for comparison, these tools were developed for traditional static educational materials and may not capture the dynamic and adaptive features of AI-generated content. Finally, we did not perform a formal qualitative or thematic analysis, meaning that nuanced impressions about clinical depth or emphasis were not systematically assessed.

### Future Directions

Future research should adopt evaluation designs that better reflect how individuals seek health information in real-world settings. Because this study used a single standardized question, future work would benefit from multiscenario approaches that incorporate sequential prompts, follow-up queries, and patient-specific contexts. Such designs may offer a more realistic understanding of how chatbots manage evolving information needs and uncertainty, particularly in complex neuroimmunological disorders such as MOGAD.

There is also a need for tools specifically developed to assess AI-generated medical content. While DISCERN, PEMAT-P, and WRR remain valuable, they were designed for static patient education materials and do not fully capture the adaptive and conversational nature of LLMs. Future metrics should evaluate hallucination risk, citation quality, internal consistency, and clinical safety. They should also integrate usability testing with patients and caregivers to clarify how tone, framing, and readability influence understanding.

Multilingual and culturally sensitive assessments represent another important direction. As chatbots expand globally, their performance across different languages, health systems, and literacy levels should be examined to identify potential disparities in access to reliable online health information. Longitudinal evaluations will also be important, as rapid updates to model architecture may lead to meaningful changes in accuracy and transparency over time.

Finally, incorporating qualitative methods such as thematic or discourse-level analysis may provide deeper insight into how chatbot responses structure complex concepts, communicate uncertainty, and present risk–benefit information. Blending qualitative insights with quantitative scoring may support the development of safer and more clinically meaningful AI tools for individuals seeking information about MOGAD and other rare neurological disorders.

### Conclusions

This study provides a multidimensional evaluation of AI chatbot responses to a patient-centered MOGAD query and demonstrates marked variability across platforms in scientific quality, understandability, transparency, and readability. Paid-access models achieved significantly higher scores in scientific quality, understandability, and citation transparency, whereas search-focused systems showed modest but statistically significant advantages in understandability, highlighting a trade-off rather than uniform superiority across model types. Importantly, all evaluated chatbots produced content exceeding recommended readability thresholds, indicating persistent barriers to linguistic accessibility, particularly for patients and caregivers navigating a rare and clinically complex disorder.

These findings underscore that strong citation practices or higher transparency scores do not necessarily guarantee clinical relevance or usability, and that current evaluation instruments may incompletely capture risks specific to AI-generated medical content. For rare diseases such as MOGAD, ensuring safe and patient-centered use of chatbots will require assessment frameworks that better account for readability, contextual accuracy, and potential retrieval-related errors, alongside traditional measures of quality and transparency.

## Supplementary material

10.2196/81720 Multimedia Appendix 1 Chatbot responses.

## Abbreviations

AHRQ: U.S. Agency for Healthcare Research and Quality
AI: artificial intelligence
CLI: Coleman-Liau Index
FKGL: Flesch-Kincaid Grade Level
ICC: intraclass correlation coefficient
LLM: large language model
MOGAD: myelin oligodendrocyte glycoprotein antibody–associated disease
PEMAT-P: Patient Education Materials Assessment Tool for Printable Material
WRR: Web Resource Rating
