# Supplementary material for: Evaluation of AI Chatbot Responses to a Standardized Patient Query on Myelin Oligodendrocyte Glycoprotein Antibody–Associated Disease: Cross-Sectional Content Analysis
Source: JMIR Med Inform. 2026 Apr 29;14:e81720. doi: 10.2196/81720 (PMC13128063; doi:10.2196/81720)
Supplement: Multimedia Appendix 1 [file medinform-v14-e81720-s001.pdf]

Chatbot 6

Myelin Oligodendrocyte Glycoprotein Antibody-Associated Disease (MOGAD) is a rare autoimmune disorder where the immune system mistakenly attacks the myelin sheath—the protective covering of nerve fibers—in the central nervous system (CNS), including the optic nerves, spinal cord, and brain. This attack leads to inflammation and demyelination, disrupting nerve signal transmission.

Symptoms of MOGAD

MOGAD presents with various neurological symptoms, which can vary in severity:

- **Optic Neuritis**: Painful vision loss, often in both eyes.
- **Transverse Myelitis**: Muscle weakness, numbness, and bladder or bowel dysfunction.
- **Acute Disseminated Encephalomyelitis (ADEM)**: More common in children, leading to confusion, seizures, and widespread brain inflammation.

These symptoms can occur as a single episode (monophasic) or recur over time (relapsing).

Treatment of MOGAD

While there is no definitive cure for MOGAD, treatments focus on managing acute attacks and preventing relapses.

Acute Attack Management:

- **High-dose Intravenous Corticosteroids**: Typically administered over 3–5 days to reduce inflammation.
- **Plasma Exchange (PLEX)**: Used if symptoms are severe or unresponsive to steroids, helping to remove harmful antibodies from the blood.
- **Intravenous Immunoglobulin (IVIG)**: An alternative or adjunct therapy, especially in pediatric cases.

Long-term Maintenance Therapy:

For patients with relapsing MOGAD, ongoing immunosuppressive treatments may be recommended:

- **Rituximab**: A monoclonal antibody that depletes B cells, reducing antibody production.
- **Azathioprine**: An immunosuppressant that inhibits lymphocyte proliferation.
- **Mycophenolate Mofetil**: Suppresses the immune response by inhibiting purine synthesis in lymphocytes.
- **Regular IVIG Infusions**: To modulate the immune system and prevent relapses.

The choice of maintenance therapy is individualized, considering factors like age, disease severity, and treatment response.

Prognosis

ChatGPT 4.5

Many patients respond well to treatment, especially when initiated promptly. However, some may experience residual neurological deficits, particularly if transverse myelitis is present at onset. Early diagnosis and tailored therapy are crucial for optimal outcomes.

References

- Cleveland Clinic: MOGAD Overview
- Mayo Clinic: MOGAD Diagnosis & Treatment
- National Organization for Rare Disorders: MOG Antibody Disease
- PubMed Central: Treatment of MOG Antibody Associated Disorders

If you have further questions or need more detailed information, feel free to ask!

Chat GPT 4.5

Chatbot 10

Myelin oligodendrocyte glycoprotein antibody-associated disease (MOGAD) is a rare autoimmune disorder that affects the central nervous system (CNS), including the optic nerves, spinal cord, and brain. In MOGAD, the immune system mistakenly targets the myelin oligodendrocyte glycoprotein (MOG), a protein located on the surface of myelin sheaths that insulate nerve fibers, leading to inflammation and demyelination. (MOGAD: What It Is, Diagnosis, Symptoms & Treatment, MOG Antibody Disease - Symptoms, Causes, Treatment | NORD)

Symptoms and Clinical Features

MOGAD can present with various neurological symptoms, depending on the areas of the CNS affected. Common manifestations include:

- **Optic neuritis**: Inflammation of the optic nerve, leading to visual disturbances or vision loss.
- **Transverse myelitis**: Inflammation of the spinal cord, causing weakness, sensory changes, or bladder dysfunction.
- **Acute disseminated encephalomyelitis (ADEM)**: Widespread inflammation in the brain and spinal cord, more common in children.

The disease course can be monophasic (a single episode) or relapsing (multiple episodes over time). Approximately half of the patients experience a relapsing course.

Diagnosis

Diagnosis of MOGAD involves detecting antibodies against MOG in the patient's serum using cell-based assays. Magnetic resonance imaging (MRI) of the brain and spinal cord, along with clinical evaluation, aids in distinguishing MOGAD from other demyelinating diseases like multiple sclerosis (MS) and neuromyelitis optica spectrum disorder (NMOSD). (Epidemiology of myelin oligodendrocyte glycoprotein antibody ...)

Treatment

Treatment strategies for MOGAD focus on managing acute attacks and preventing future relapses:

- **Acute attacks**:
    - **High-dose corticosteroids**: Intravenous methylprednisolone is commonly used to reduce inflammation during acute episodes.
    - **Plasma exchange (PLEX)**: Considered if patients do not respond adequately to corticosteroids.
- **Relapse prevention**:
    - For patients with a relapsing course, long-term immunosuppressive therapy may be initiated. Options include oral corticosteroids, azathioprine, mycophenolate mofetil, or rituximab.

It's important to note that certain treatments effective in MS, such as interferon-beta, may not be beneficial in MOGAD and could potentially worsen the condition.

ChatGPT 4

Prognosis

The prognosis for MOGAD varies. Many patients recover well from initial attacks, especially with prompt treatment. However, some may experience residual neurological deficits, particularly if treatment is delayed or if relapses occur. Regular monitoring and early intervention are crucial to improve long-term outcomes.

For more detailed information, you can refer to resources like the Cleveland Clinic's overview on MOGAD. (MOGAD: What It Is, Diagnosis, Symptoms & Treatment) (Myelin Oligodendrocyte Antibody Disease - Cleveland Clinic)

ChatGPT 4

Chatbot 2

Hi there! I'd be happy to explain MOGAD and its treatments.

MOGAD stands for Myelin Oligodendrocyte Glycoprotein Antibody-Associated Disease. It's a rare autoimmune disorder where antibodies target the myelin oligodendrocyte glycoprotein (MOG) found on the surface of myelin sheaths in the central nervous system. This leads to inflammation and damage to the myelin, affecting signal transmission in the nervous system.

MOGAD is relatively newly recognized as distinct from other demyelinating conditions like multiple sclerosis (MS) and neuromyelitis optica spectrum disorder (NMOSD).

Common treatments for MOGAD include:

1. Acute treatments:
   - High-dose corticosteroids (like methylprednisolone)
   - Plasma exchange (PLEX)
   - Intravenous immunoglobulin (IVIG)
2. Long-term/preventative treatments:
   - Rituximab
   - Mycophenolate mofetil
   - Azathioprine
   - Monthly IVIG
   - Tocilizumab

Since you asked for real references, I should clarify that I don't have the ability to search the internet or access specific scientific papers to cite. My knowledge has a cutoff date of October 2024, and while I'm familiar with the condition and treatments, I can't provide direct citations to current research papers or guidelines.

For the most up-to-date information and specific references, I'd recommend consulting resources like PubMed, journals focusing on neuroimmunology, or organizations like the National Multiple Sclerosis Society, which often include information on related conditions like MOGAD.

Would you like me to go into more detail about how specific treatments work, or would you prefer information about symptoms or diagnosis?

Claude Free

Chatbot 9

Hi there! I'd be happy to explain MIGAD and MOGAD, as well as their treatments.

MIGAD (Myelin Oligodendrocyte Glycoprotein Antibody-associated Disease) is actually more commonly referred to as MOGAD (MOG Antibody-associated Disease). These terms refer to the same condition, which is a relatively newly recognized autoimmune disorder of the central nervous system characterized by antibodies against myelin oligodendrocyte glycoprotein (MOG).

MOGAD is distinct from multiple sclerosis (MS) and aquaporin-4 antibody-positive neuromyelitis optica spectrum disorder (AQP4-NMOSD), though they share some clinical features.

Key Features of MOGAD:

- Affects both children and adults
- Can present as optic neuritis, transverse myelitis, acute disseminated encephalomyelitis (ADEM), or brainstem encephalitis
- Often has a relapsing course, though some patients experience monophasic illness
- Diagnosis confirmed by serum MOG-IgG antibody testing

Treatment of MOGAD:

MOGAD treatment typically involves:

1. **Acute treatment**:
   - High-dose intravenous methylprednisolone
   - Plasma exchange for severe or steroid-unresponsive cases
   - Intravenous immunoglobulin (IVIG) in some cases
2. **Maintenance/preventive treatment**:
   - Oral corticosteroids (often with prolonged taper)
   - Immunosuppressants (azathioprine, mycophenolate mofetil, rituximab)
   - Monthly IVIG in some cases
   - Regular monitoring for relapses

References:

1. Wynford-Thomas R, Jacob A, Tomassini V. Neurological update: MOG antibody disease. J Neurol. 2019;266(5):1280-1286.
2. Jarius S, Paul F, Aktas O, et al. MOG encephalomyelitis: international recommendations on diagnosis and antibody testing. J Neuroinflammation. 2018;15(1):134.
3. Jurynczyk M, Messina S, Woodhall MR, et al. Clinical presentation and prognosis in MOG-antibody disease: a UK study. Brain. 2017;140(12):3128-3138.

Claude 3.7

4. Ramanathan S, Mohammad S, Tantsis E, et al. Clinical course, therapeutic responses and outcomes in relapsing MOG antibody-associated demyelination. J Neurol Neurosurg Psychiatry. 2018;89(2):127-137.
5. Cobo-Calvo A, Ruiz A, Maillart E, et al. Clinical spectrum and prognostic value of CNS MOG autoimmunity in adults: The MOGADOR study. Neurology. 2018;90(21):e1858-e1869.

Would you like more specific information about any particular aspect of MOGAD, such as its pathophysiology, diagnostic criteria, or specific treatment regimens?

Claude 3.7

Chatbot 1

MOGAD, or Myelin Oligodendrocyte Glycoprotein Antibody-Associated Disease, is a rare autoimmune condition where the immune system mistakenly attacks the myelin, the protective covering of nerves in the central nervous system. This can lead to symptoms such as vision loss, muscle weakness, and coordination issues.

Treatment for MOGAD typically focuses on managing acute attacks, preventing relapses, and addressing symptoms. Common approaches include:

- **Acute Attack Management**: High-dose corticosteroids are often used to reduce inflammation during an attack. In severe cases, plasma exchange or intravenous immunoglobulin (IVIG) may be employed.
- **Preventative Treatment**: For patients with relapsing MOGAD, long-term therapies may include immunosuppressive medications to reduce the risk of future attacks. Research is ongoing to explore new treatment options.

You can find more detailed information on MOGAD and its treatment [here](https://my.clevelandclinic.org/health/diseases/myelin-oligodendrocyte-glycoprotein-antibody-disease-mogad) and [here](https://www.mayoclinic.org/diseases-conditions/mogad/diagnosis-treatment/drc-20560477). Let me know if you'd like to dive deeper into any specific aspect!

Copilot

Chatbot 8

What is MOGAD?

MOGAD stands for **Myelin Oligodendrocyte Glycoprotein Antibody-Associated Disease**, an autoimmune disorder of the central nervous system (CNS). It is characterized by inflammation and demyelination (damage to the protective myelin sheath around nerves) due to autoantibodies targeting the **MOG protein**, which is found on the surface of myelin in the CNS.

MOGAD is distinct from **multiple sclerosis (MS)** and **neuromyelitis optica spectrum disorder (NMOSD)** but shares some overlapping features.

Key Clinical Features of MOGAD

- **Optic neuritis** (inflammation of the optic nerve, causing vision loss)
- **Transverse myelitis** (spinal cord inflammation, leading to weakness/sensory loss)
- **Acute disseminated encephalomyelitis (ADEM)** (brain inflammation, more common in children)
- **Brainstem or cortical encephalitis** (rare but possible)

Diagnosis of MOGAD

- **MOG antibody testing** (cell-based assay, **CBA**, is the gold standard)
- **MRI findings**: Lesions in the optic nerves, spinal cord, or brain, often with a distinct pattern
- **Exclusion of MS/NMOSD** (AQP4 antibody-negative)

Treatment of MOGAD

MOGAD is typically treated with **immunotherapy**, especially during acute attacks and for relapse prevention.

1. Acute Attacks (High-Dose Steroids)

- **First-line**: **Intravenous methylprednisolone (IVMP)** (1g/day for 3–5 days)
- If severe or unresponsive: **Plasma exchange (PLEX)** or **IV immunoglobulin (IVIG)**

2. Long-Term Immunotherapy (Relapse Prevention)

- **First-line**:
  - **Oral corticosteroids** (e.g., prednisone taper over months)
  - **IVIG** (especially in children)
- **Second-line (for frequent relapses)**:
  - **Rituximab** (anti-CD20 monoclonal antibody)
  - **Mycophenolate mofetil**
  - **Azathioprine**
  - **Tocilizumab** (IL-6 inhibitor, emerging evidence)

3. Emerging Therapies

DeepSeek

- **Satralizumab** (anti-IL-6, under investigation)
- **Eculizumab** (complement inhibitor, rare cases)

Prognosis

- MOGAD can be **monophasic** (single attack) or **relapsing** (~50% of cases).
- Early treatment improves outcomes.
- Children often have a better prognosis than adults.

Key References (Peer-Reviewed Studies & Guidelines)

1. **Jarius et al. (2016)** – MOGAD clinical spectrum (*Journal of Neuroinflammation*).
2. **Reindl et al. (2020)** – MOG antibody testing & treatment (*Nature Reviews Neurology*).
3. **López-Chiriboga et al. (2018)** – Rituximab efficacy in MOGAD (*Neurology: Neuroimmunology & Neuroinflammation*).
4. **Marignier et al. (2021)** – International MOGAD consensus guidelines (*Lancet Neurology*).

Would you like specific details on a particular aspect of MOGAD (e.g., pediatric cases, MRI findings, or latest trials)?

Note: Always consult a neurologist for personalized diagnosis and treatment.

DeepSeek

Chatbot 7

Okay, let's break down what MOGAD is and how it's treated.

What is MOGAD?

1. **Full Name:** MOGAD stands for **Myelin Oligodendrocyte Glycoprotein Antibody-Associated Disease**.
2. **Nature of the Disease:** It is an autoimmune inflammatory disorder affecting the central nervous system (CNS), which includes the brain, spinal cord, and optic nerves.
3. **Mechanism:** In MOGAD, the body's immune system mistakenly produces antibodies that target and attack a protein called Myelin Oligodendrocyte Glycoprotein (MOG). This MOG protein is found on the surface of myelin (the protective sheath covering nerve fibers) and oligodendrocytes (the cells that produce and maintain myelin in the CNS).
4. **Consequences:** This autoimmune attack causes inflammation and demyelination (damage or loss of the myelin sheath). This damage disrupts the normal transmission of nerve signals.
5. **Symptoms:** The symptoms of MOGAD vary depending on which part of the CNS is affected. Common presentations include:
    - **Optic Neuritis:** Inflammation of the optic nerve, causing vision loss, eye pain, and sometimes color vision changes. This is a very common first symptom.
    - **Transverse Myelitis:** Inflammation across a section of the spinal cord, leading to weakness, numbness, tingling in the limbs, back pain, and often bladder/bowel dysfunction.
    - **Acute Disseminated Encephalomyelitis (ADEM)-like presentation:** Inflammation primarily in the brain and/or spinal cord, often causing a wide range of neurological symptoms like confusion, drowsiness, weakness, vision changes, and sometimes seizures. This is more common in children.
    - **Brainstem or Cerebellar Symptoms:** Less commonly, inflammation can affect the brainstem or cerebellum, leading to issues like double vision, coordination problems, or difficulty swallowing.
6. **Distinction from other diseases:** MOGAD was once often confused with Multiple Sclerosis (MS) or Neuromyelitis Optica Spectrum Disorder (NMOSD). However, the discovery of the specific anti-MOG antibody has established it as a distinct condition. While symptoms can overlap, the underlying cause (specific antibody target) and often the disease course and response to treatment differ. For instance, MOGAD typically doesn't lead to the same level of progressive disability between attacks as sometimes seen in MS, and the antibody target is different from the Aquaporin-4 antibody often found in NMOSD.

How is MOGAD Treated?

Treatment for MOGAD focuses on two main goals: managing acute attacks (relapses) and preventing future attacks (long-term maintenance therapy).

1. **Treating Acute Attacks (Relapses):**
    - **Goal:** To quickly reduce inflammation and minimize damage to the CNS.

Gemini 2.5 Pro

    - **First-line Treatment:** High-dose intravenous corticosteroids (e.g., methylprednisolone) are typically used for several days, often followed by a slow taper of oral corticosteroids.
    - **Second-line Treatment:** If steroids are not effective enough or cannot be used, other options include:
        - **Plasma Exchange (PLEX):** A procedure to remove the harmful antibodies from the blood.
        - **Intravenous Immunoglobulin (IVIg):** Administration of pooled antibodies from healthy donors, which can help modulate the immune system.
2. **Long-Term Maintenance Therapy (Preventing Relapses):**
    - **Goal:** To suppress the immune system long-term to prevent future attacks. This is usually recommended for patients who have had recurrent attacks or a particularly severe initial attack.
    - **Options:** There are currently no therapies specifically FDA-approved *for* MOGAD, so treatments are often used "off-label" based on data from observational studies and experience with other autoimmune neurological diseases. Common maintenance therapies include:
        - **Rituximab:** An antibody therapy that depletes B-cells (the immune cells that produce antibodies).
        - **Mycophenolate Mofetil:** An oral immunosuppressant drug.
        - **Azathioprine:** Another oral immunosuppressant drug.
        - **Intravenous Immunoglobulin (IVIg):** Can also be used regularly as a maintenance therapy.
        - **Low-dose Oral Corticosteroids:** Sometimes used long-term, but efforts are usually made to minimize their use due to side effects.
    - **Important Note:** Some treatments used for MS (like interferons or glatiramer acetate) are generally considered ineffective or may even worsen MOGAD, highlighting the importance of accurate diagnosis.
3. **Symptomatic Management:** Addressing residual symptoms from nerve damage (like pain, fatigue, bladder problems, spasticity) is also a crucial part of managing MOGAD. This may involve medications, physical therapy, and other supportive measures.

Diagnosis and treatment decisions for MOGAD should always be made by a neurologist or a specialist experienced in neuroimmunological disorders.

Kaynaklar ve ilgili içerik

Myelin oligodendrocyte glycoprotein antibody-associated...
www.mayoclinic.org

MOGAD: What It Is, Diagnosis, Symptoms & Treatment - Clevelan...
my.clevelandclinic.org

Myelin oligodendrocyte glycoprotein antibody-associate
www.kuh.ku.edu.tr

Gemini 2.5 Pro

Chatbot 5

MOGAD (Myelin Oligodendrocyte Glycoprotein Antibody-Associated Disease) is a recently recognized autoimmune disorder of the central nervous system (CNS) that is distinct from multiple sclerosis (MS) and neuromyelitis optica spectrum disorder (NMOSD). In MOGAD, the body's immune system mistakenly attacks the myelin oligodendrocyte glycoprotein (MOG), a protein found on the surface of myelin, the protective sheath that covers nerve fibers in the brain, spinal cord, and optic nerves. This attack leads to inflammation and demyelination, disrupting nerve signal transmission and causing various neurological symptoms.

Key features of MOGAD:

- **Autoimmune Basis:** MOGAD is characterized by the presence of autoantibodies (MOG-IgG) that target the MOG protein.
- **Clinical Overlap:** Clinically, MOGAD can overlap with other demyelinating diseases like MS and NMOSD, making diagnosis challenging. However, it has distinct features.
- **Common Presentations:** The most common presentations include:
  - **Optic Neuritis:** Inflammation of the optic nerve, often bilateral and severe, leading to vision loss and eye pain.
  - **Transverse Myelitis:** Inflammation of the spinal cord, causing weakness, numbness, paralysis, and bowel/bladder dysfunction.
  - **ADEM-like Encephalomyelitis:** Inflammation of the brain, particularly in children, leading to confusion, behavioral changes, seizures, and coordination problems.
- **Relapsing Course:** MOGAD can be monophasic (a single episode) or relapsing (multiple attacks). Relapses are common, especially in the first few years after onset.
- **Age Distribution:** While it can occur at any age, MOGAD is slightly more common in children and young adults.

Diagnosis of MOGAD:

The diagnosis of MOGAD typically involves a combination of clinical evaluation, magnetic resonance imaging (MRI), and laboratory testing:

1. **Core Clinical Demyelinating Event:** The patient must have one or more typical demyelinating events such as optic neuritis, transverse myelitis, ADEM, or brainstem/cerebellar syndromes.
2. **Positive MOG-IgG Antibody Test:** The presence of MOG antibodies in the blood is crucial. Testing should be performed using a cell-based assay, which is considered more sensitive and specific. The timing of the test in relation to the clinical attack can influence the results.
3. **Exclusion of Other Diagnoses:** Other demyelinating diseases, particularly MS and AQP4-positive NMOSD, need to be ruled out based on clinical, radiological, and laboratory findings.

Supportive clinical or MRI features can further aid in the diagnosis, especially in cases with low-positive or negative MOG-IgG titers but strong clinical suspicion. These features include

Gemini Advanced

bilateral simultaneous optic neuritis, longitudinally extensive optic nerve or spinal cord lesions, and specific brain MRI findings.

Treatment of MOGAD:

There is currently no cure for MOGAD, and treatment focuses on:

1. **Treating Acute Attacks:** Prompt treatment of acute relapses is essential for the most complete recovery. Options include:
   - **High-dose intravenous corticosteroids:** To reduce inflammation.
   - **Plasma exchange (PLEX):** A procedure to remove antibodies from the blood, often used in severe attacks or when steroids are insufficient.
   - **Intravenous immunoglobulin (IVIG):** Another immunomodulatory therapy that can be effective in some cases.
2. **Preventing Future Attacks (Relapses):** For patients with relapsing MOGAD, long-term immunosuppressive or immunomodulatory treatments are often considered. However, there is no definitive consensus on the optimal maintenance therapy. Options used include:
   - **Oral immunosuppressants:** Such as azathioprine, mycophenolate mofetil, and low-dose prednisone, which suppress the immune system.
   - **Intravenous therapies:** Like rituximab (a B-cell depleting agent) and IVIG, which can also help prevent relapses.
3. **Symptomatic Management:** Various medications and therapies are used to manage specific symptoms such as pain, fatigue, seizures, and bowel/bladder dysfunction. Physical and occupational therapy can help with rehabilitation after attacks.

References:

While specific URLs are not to be provided, the information above is based on current medical literature and expert consensus, including publications in journals such as *The Lancet Neurology*, *Neurology*, *Multiple Sclerosis and Related Disorders*, and *Journal of Neuroinflammation*. These resources provide detailed diagnostic criteria, treatment strategies, and long-term outcomes in MOGAD. You can find these by searching for "MOGAD diagnosis criteria", "MOGAD treatment", and "myelin oligodendrocyte glycoprotein antibody-associated disease" in medical databases like PubMed and Google Schola

Gemini Advanced

Chatbot 4

Myelin Oligodendrocyte Glycoprotein Antibody-Associated Disease (MOGAD) is a rare autoimmune disorder where the immune system attacks the myelin oligodendrocyte glycoprotein (MOG), a protein on the myelin sheath of nerves in the central nervous system (CNS). This leads to inflammation and demyelination, causing symptoms like vision loss, muscle weakness, confusion, seizures, or spinal cord issues, depending on the affected area. MOGAD is distinct from multiple sclerosis (MS) and neuromyelitis optica spectrum disorder (NMOSD), though symptoms may overlap. It can be monophasic (single attack) or relapsing and often follows a viral infection in 37–70% of cases. It affects both children and adults, with children more likely to present with acute disseminated encephalomyelitis (ADEM) and adults with optic neuritis or transverse myelitis.

Diagnosis
Diagnosis relies on:
- **Clinical symptoms**: Optic neuritis (unilateral or bilateral), transverse myelitis, ADEM, or encephalitis.
- **MOG antibody testing**: Detection of MOG-IgG in serum using cell-based assays (CBA) is critical for accurate diagnosis. Testing in cerebrospinal fluid (CSF) is less common but may show elevated white cells or protein during attacks.
- **MRI**: Shows lesions in the optic nerve, spinal cord, or brain, often with longitudinally extensive optic nerve enhancement or perivenous demyelination patterns, distinct from MS.
- **Exclusion of other conditions**: MS, NMOSD (aquaporin-4 antibody-positive), or other causes must be ruled out, as low-titer MOG antibodies can occasionally appear in healthy individuals or other diseases.

Treatment
There’s no cure for MOGAD, but treatments aim to manage acute attacks, reduce symptoms, and prevent relapses. Most evidence comes from observational studies, as randomized controlled trials are limited due to the disease’s rarity.

Acute Attack Treatment
- **High-dose corticosteroids**: Intravenous methylprednisolone (e.g., 1 g/day for 3–5 days) is the first-line treatment to reduce inflammation and reverse symptoms quickly. Oral prednisone may follow for 1–4 weeks. Side effects include mood swings, increased blood pressure, and fluid retention.‡web:15ᒣ[](https://my.clevelandclinic.org/health/diseases/myelin-oligodendrocyte-glycoprotein-antibody-disease-mogad)
- **Plasma exchange (PLEX)**: Used if steroids are insufficient, PLEX removes MOG antibodies from the blood. It’s effective for severe attacks but requires specialized equipment.‡web:12ᒣ[](https://www.ncbi.nlm.nih.gov/pmc/articles/PMC7795410/)
- **Intravenous immunoglobulin (IVIG)**: IVIG replaces harmful antibodies with donor antibodies, often used in children or when steroids fail. It’s effective for acute attacks and may reduce relapse risk. Side effects include headache or allergic

Grok3

reactions.‡web:127[](https://my.clevelandclinic.org/health/diseases/myelin-oligodendrocyte-glycoprotein-antibody-disease-mogad)

Maintenance Therapy (Relapse Prevention)
Maintenance therapy is typically reserved for relapsing MOGAD or severe initial attacks causing permanent disability. Options include:
- **Oral immunosuppressants**: Azathioprine, mycophenolate mofetil, or methotrexate suppress the immune system to prevent attacks. These take months to become effective and carry risks like infection or rash.‡web:247[](https://my.clevelandclinic.org/departments/neurological/depts/multiple-sclerosis/ms-approaches/myelin-oligodendrocyte-glycoprotein-antibody-disease)
- **Rituximab**: A monoclonal antibody targeting B cells, reducing antibody production. Observational data show it reduces relapse rates, though some patients remain refractory.‡web:127[](https://my.clevelandclinic.org/departments/neurological/depts/multiple-sclerosis/ms-approaches/myelin-oligodendrocyte-glycoprotein-antibody-disease)
- **IVIG**: Maintenance IVIG (monthly or subcutaneous) has shown efficacy in preventing relapses, particularly in adults. A 2022 study found it significantly reduced relapse risk. (Chen JJ et al., JAMA Neurol. 2022;79(5):518-525. doi:10.1001/jamaneurol.2022.0489)[](https://rarediseases.org/rare-diseases/mog-antibody-disease/)
- **IL-6 receptor blockers**: Tocilizumab, which blocks IL-6 signaling, has been effective in rituximab-refractory cases. A 2021 study confirmed its safety and efficacy. (Ringelstein M et al., Neurol Neuroimmunol Neuroinflamm. 2021;9(1):e1100. doi:10.1212/NXI.0000000000001100)[](https://rarediseases.org/rare-diseases/mog-antibody-disease/)
- **Emerging therapies**: Ongoing trials are evaluating satralizumab (NCT05271409), azathioprine (NCT05349006), and Fc receptor antibodies like rozanolixizumab.[](https://my.clevelandclinic.org/departments/neurological/depts/multiple-sclerosis/ms-approaches/myelin-oligodendrocyte-glycoprotein-antibody-disease)

Rehabilitation
For persistent deficits (e.g., vision loss or mobility issues), rehabilitation programs help patients regain independence through physical or occupational therapy.[](https://my.clevelandclinic.org/health/diseases/myelin-oligodendrocyte-glycoprotein-antibody-disease-mogad)

Prognosis
Complete recovery from the first attack is common, especially in children, but relapsing MOGAD increases the risk of permanent deficits, such as vision loss or motor impairment. Regular monitoring with MRI and clinical exams is crucial, as T2-lesions often resolve within 6–12 months, supporting a MOGAD diagnosis.[](https://x.com/JNNP_BMJ/status/1911732456840974722)

Challenges and Future Directions

- **Limited trial data**: The rarity of MOGAD complicates large-scale trials, so treatments rely on NMOSD/MS protocols or observational data.[](https://www.ncbi.nlm.nih.gov/pmc/articles/PMC7795410/)
- **Diagnostic accuracy**: False positives in low-titer MOG antibody tests require careful interpretation alongside clinical and MRI findings.[](https://pmc.ncbi.nlm.nih.gov/articles/PMC9247462/)
- **Ongoing research**: Trials and registries (e.g., Siegel Rare Neuroimmune Association, The MOG Project) are advancing understanding of MOGAD's pathophysiology and optimal therapies.[](https://www.childneurologyfoundation.org/disorder/myelin-oligodendrocyte-glycoprotein-antibody-disease-mogad/)

References
1. Banwell B et al. Diagnosis of myelin oligodendrocyte glycoprotein antibody-associated disease: International MOGAD panel proposed criteria. Lancet Neurol. 2023;22(3):268-282. doi:10.1016/S1474-4422(22)00431-8[](https://www.childneurologyfoundation.org/disorder/myelin-oligodendrocyte-glycoprotein-antibody-disease-mogad/)
2. Sechi E et al. Myelin Oligodendrocyte Glycoprotein Antibody-Associated Disease (MOGAD): A Review of Clinical and MRI Features, Diagnosis, and Management. Front Neurol. 2022;13:885218. doi:10.3389/fneur.2022.885218[](https://www.childneurologyfoundation.org/disorder/myelin-oligodendrocyte-glycoprotein-antibody-disease-mogad/)
3. Chen JJ et al. Association of maintenance intravenous immunoglobulin with prevention of relapse in adult myelin oligodendrocyte glycoprotein antibody-associated disease. JAMA Neurol. 2022;79(5):518-525. doi:10.1001/jamaneurol.2022.0489[](https://rarediseases.org/rare-diseases/mog-antibody-disease/)
4. Ringelstein M et al. Interleukin-6 receptor blockade in treatment-refractory MOG-IgG–associated disease and neuromyelitis optica spectrum disorders. Neurol Neuroimmunol Neuroinflamm. 2021;9(1):e1100. doi:10.1212/NXI.0000000000001100[](https://rarediseases.org/rare-diseases/mog-antibody-disease/)
5. Marignier R et al. Myelin-oligodendrocyte glycoprotein antibody-associated disease. Lancet Neurol. 2021;20(9):762-772. doi:10.1016/S1474-4422(21)00218-0[](https://www.thelancet.com/journals/laneur/article/PIIS1474-4422%2821%2900218-0/abstract)

For more resources, visit Siegel Rare Neuroimmune Association (wearesrna.org) or The MOG Project (mogproject.org). If you have specific symptoms or need personalized advice, consult a neurologist specializing in neuroimmunology.[](https://www.childneurologyfoundation.org/disorder/myelin-oligodendrocyte-glycoprotein-antibody-disease-mogad/)

Grok 3

Chatbot 3

What is MOGAD?

Myelin oligodendrocyte glycoprotein antibody-associated disease (MOGAD) is a rare, autoimmune, inflammatory disorder of the central nervous system (CNS) in which the immune system mistakenly attacks a protein called myelin oligodendrocyte glycoprotein (MOG) found on the myelin sheath covering nerve fibers in the brain, spinal cord, and optic nerves[2][4][6][12]. This immune attack leads to demyelination, causing inflammation and damage to the optic nerves (optic neuritis), spinal cord (myelitis), and sometimes the brain and brainstem[4][6][9]. Symptoms can include vision loss, muscle weakness, paralysis, sensory changes, bladder or bowel dysfunction, seizures, and confusion[4][6][11].

MOGAD is distinct from multiple sclerosis (MS) and aquaporin-4 antibody-positive neuromyelitis optica spectrum disorder (NMOSD), although symptoms can overlap[3][7][12].

How is MOGAD Treated?

There is currently no cure for MOGAD, but treatment focuses on three main goals: rapidly treating acute attacks, preventing future relapses, and managing symptoms[5][6][7]. The summary table you provided aligns with current expert recommendations and can be explained as follows:

Acute Attack Treatment

- **Main Options:** High-dose corticosteroids, plasma exchange (PE or PLEX), intravenous immunoglobulin (IVIg)[1][4][5][7].
- **Purpose:** Reduce inflammation and speed up recovery after an attack.
  - High-dose corticosteroids (given orally or intravenously) are the first-line treatment to quickly reduce nerve inflammation.
  - Plasma exchange is used if symptoms are severe or do not respond to steroids, as it removes harmful antibodies from the blood.
  - IVIg is another option, especially in children or when other treatments are not effective[5].

Maintenance/Relapse Prevention

- **Main Options:** Immunosuppressive therapies such as rituximab, mycophenolate mofetil, azathioprine, IVIg, and prednisolone[1][4][5][7].

Perplexity Pro

- **Purpose:** Prevent future attacks or relapses.
  - These medications suppress the immune system to reduce the risk of new inflammatory episodes.
  - Maintenance therapy is generally considered for patients who have had multiple attacks (relapsing MOGAD) or severe initial symptoms[5][7].
  - Ongoing clinical trials are also exploring new treatments, such as satralizumab and rozanolixizumab, which target specific immune pathways[8].

Symptom Management

- **Main Options:** Symptom-specific medications, rehabilitation therapies[1][5].
- **Purpose:** Improve quality of life after attacks.
  - This may include physical and occupational therapy for muscle weakness or paralysis, antiseizure medications if seizures occur, and medications for pain, fatigue, or bladder/bowel symptoms[5].

Treatment Summary Table

Treatment Phase	Main Options	Purpose
Acute Attack	High-dose corticosteroids, PE, IVIg	Reduce inflammation, speed recovery
Maintenance/Relapse	Rituximab, mycophenolate, azathioprine, IVIg, prednisolone	Prevent future attacks/ relapses
Symptom Management	Symptom-specific medications, rehab	Improve quality of life after attacks

Key Points

- MOGAD is a rare autoimmune CNS disorder distinct from MS and NMOSD.
- Treatment is tailored to the phase of the disease: acute attacks, relapse prevention, and symptom management.
- Early and aggressive treatment of attacks is crucial for the best recovery.
- Long-term immunosuppression may be needed for those with relapsing disease.
- Symptom management and rehabilitation are important for maintaining quality of life[1][4][5][7].

If you have questions about a specific treatment or need more detail on any aspect, please let me know!

Sources
[1] image.jpg https://pplx-res.cloudinary.com/image/upload/v1744910661/user_uploads/oejkywfZWGOdDrO/image.jpg

[2] MOGAD: What It Is, Diagnosis, Symptoms & Treatment https://my.clevelandclinic.org/health/diseases/myelin-oligodendrocyte-glycoprotein-antibody-disease-mogad

[3] Myelin Oligodendrocyte Glycoprotein Antibody-Associated Disease ... https://pmc.ncbi.nlm.nih.gov/articles/PMC9247462/

[4] MOG Antibody Disease - Symptoms, Causes, Treatment | NORD https://rarediseases.org/rare-diseases/mog-antibody-disease/

[5] Myelin oligodendrocyte glycoprotein antibody-associated disease ... https://www.mayoclinic.org/diseases-conditions/mogad/diagnosis-treatment/drc-20560477

[6] Myelin oligodendrocyte glycoprotein antibody-associated disease ... https://www.mayoclinic.org/diseases-conditions/mogad/symptoms-causes/syc-20560476

[7] A Review of Clinical and MRI Features, Diagnosis, and Management https://pubmed.ncbi.nlm.nih.gov/35785363/

[8] Evaluating the Treatment Pipeline for MOGAD - NeurologyLive https://www.neurologylive.com/view/evaluating-treatment-pipeline-for-mogad

[9] Myelin Oligodendrocyte Glycoprotein Antibody Disease https://www.chop.edu/conditions-diseases/myelin-oligodendrocyte-glycoprotein-antibody-disease

[10] MOG antibody disease - Wikipedia https://en.wikipedia.org/wiki/MOG_antibody_disease

[11] Myelin oligodendrocyte glycoprotein antibody-associated disease ... https://www.amerikanhastanesi.org/mayo-clinic-care-network/mayo-clinic-health-information-library/diseases-conditions/myelin-oligodendrocyte-glycoprotein-antibody-associated-disease-mogad

[12] Myelin oligodendrocyte glycoprotein antibody disorders (MOGAD) https://www.msaustralia.org.au/nmosd-and-mogad/myelin-oligodendrocyte-glycoprotein-antibody-disorders/

[13] What is MOGAD? | MOG Antibody Disease | SRNA https://wearesrna.org/living-with-myelitis/disease-information/mog-antibody-disease/

[14] MOGAD: A comprehensive review of clinicoradiological features ... https://www.sciencedirect.com/science/article/pii/S1568997224001848

[15] MOG Antibody Disease - Symptoms, Causes, Treatment | NORD https://rarediseases.org/rare-diseases/mog-antibody-disease/

[16] Myelin oligodendrocyte glycoprotein antibody-associated disease ... https://radiopaedia.org/articles/myelin-oligodendrocyte-glycoprotein-antibody-associated-disease-mogad

[17] MOGAD Disorder and Multiple Sclerosis | National MS Society https://www.nationalmssociety.org/understanding-ms/what-is-ms/related-conditions/mogad

[18] Pediatric Myelin Oligodendrocyte Glycoprotein Antibody-Associated ... https://eyewiki.org/Pediatric_Myelin_Oligodendrocyte_Glycoprotein_Antibody-Associated_Disease_(MOGAD)

[19] The monad and the nomad: medical microbiology and the politics ... https://journals.sagepub.com/doi/pdf/10.1177/1474474010379304

perplexity pro

[20] Discovery of role of Monad in suppressing spread of breast cancer ... https://resou.osaka-u.ac.jp/en/research/2013/20130704_1

[21] The monad and the nomad: medical microbiology and the politics ... https://journals.sagepub.com/doi/abs/10.1177/1474474010379304

[22] Monad Health | Best in class healthcare services https://www.monadhealth.com

[23] Myelin oligodendrocyte glycoprotein antibody-associated disease ... https://www.kuh.ku.edu.tr/mayo-clinic-care-network/mayo-clinic-health-information-library/diseases-conditions/myelin-oligodendrocyte-glycoprotein-antibody-associated-disease-mogad

[24] Monad Pharmaceuticals Company - Medindia https://www.medindia.net/drugs/manufacturers/monad-pharmaceuticals.htm

[25] MOG Antibody Disease (MOGAD) | Prognosis & Management | SRNA https://wearesrna.org/living-with-myelitis/disease-information/mog-antibody-disease/prognosis-management/

[26] MOGAD: What It Is, Diagnosis, Symptoms & Treatment https://my.clevelandclinic.org/health/diseases/myelin-oligodendrocyte-glycoprotein-antibody-disease-mogad

[27] Optic Neuritis: NMOSD and MOGAD Treatment Evolves https://www.aao.org/eyenet/article/nmosd-and-mogad-treatment-evolves

[28] Monoclonal Antibody Therapies Beyond Complement for NMOSD ... https://pmc.ncbi.nlm.nih.gov/articles/PMC9294102/

[29] E.U. paediatric MOG consortium consensus: Part 5 – Treatment of ... https://www.sciencedirect.com/science/article/pii/S1090379820301999

[30] FAQs | Monad Doc - Marc Winiecki https://www.monaddoc.com/about-5

[31] Monad NP 100mg/500mg Tablet - 1mg https://www.1mg.com/drugs/monad-np-100mg-500mg-tablet-468964

[32] TMJ disorders - Diagnosis and treatment - Mayo Clinic https://www.mayoclinic.org/diseases-conditions/tmj/diagnosis-treatment/drc-20350945

[33] Mondor disease (breast) | Radiology Reference Article - Radiopaedia https://radiopaedia.org/articles/mondor-disease-breast

[34] Multicentric osteolysis, nodulosis, and arthropathy - MedlinePlus https://medlineplus.gov/genetics/condition/multicentric-osteolysis-nodulosis-and-arthropathy/

Perplexity Pro
